# Development of a single, practical measure of surgical site infection (SSI) for patient report or observer completion

**DOI:** 10.1177/1757177416689724

**Published:** 2017-02-01

**Authors:** Rhiannon C Macefield, Barnaby C Reeves, Thomas K Milne, Alexandra Nicholson, Natalie S Blencowe, Melanie Calvert, Kerry NL Avery, David E Messenger, Richard Bamford, Thomas D Pinkney, Jane M Blazeby

**Affiliations:** 1 School of Social and Community Medicine, University of Bristol, Bristol, UK; 2 School of Clinical Sciences, University of Bristol, Bristol, UK; 3 University Hospitals Bristol NHS Foundation Trust, Bristol, UK; 4 Severn and Peninsula Audit and Research Collaborative for Surgeons (SPARCS), UK; 5 Centre for Patient Reported Outcomes Research, University of Birmingham, Birmingham, UK; 6 Institute of Applied Health Research, University of Birmingham, Birmingham, UK; 7 Academic Department of Surgery, Queen Elizabeth Hospital, University of Birmingham, UK

**Keywords:** Outcome measure, post-discharge wound assessment, questionnaire development, surgical site infection (SSI), surgical wound, surveillance, wound healing

## Abstract

**Background:**

Surgical site infections (SSIs) are the third most common hospital-associated infection and can lead to significant patient morbidity and healthcare costs. Identification of SSIs is key to surveillance and research but reliable assessment is challenging, particularly after hospital discharge when most SSIs present. Existing SSI measurement tools have limitations and their suitability for post-discharge surveillance is uncertain.

**Aims:**

This study aimed to develop a single measure to identify SSI after hospital discharge, suitable for patient or observer completion.

**Methods:**

A three-phase mixed methods study was undertaken: Phase 1, an analysis of existing tools and semi-structured interviews with patients and professionals to establish the content of the measure; Phase 2, development of questionnaire items suitable for patients and professionals; Phase 3, pre-testing the single measure to assess acceptability and understanding to both stakeholder groups. Interviews and pre-testing took place over 12 months in 2014–2015 with patients and professionals from five specialties recruited from two UK hospital Trusts.

**Findings:**

Analyses of existing tools and interviews identified 19 important domains for assessing SSIs. Domains were developed into provisional questionnaire items. Pre-testing and iterative revision resulted in a final version with 16 items that were understood and easily completed by patients and observers (healthcare professionals).

**Conclusion:**

A single patient and observer measure for post-discharge SSI assessment has been developed. Further testing of the validity, reliability and accuracy of the measure is underway.

## Introduction

Surgical site infection (SSI) is the third most common healthcare-associated infection (HCAI) to occur after surgery (Smyth et al., 2008). Occurrence is associated with multiple factors, including patient co-morbidity, wound site, degree of contamination and whether surgery is performed in the elective or emergency setting. Incidence may be as great as 40% (Anthony et al., 2011), being highest in unplanned procedures involving the gastrointestinal tract (Pinkney et al., 2013; Public Health England, 2014). SSI leads to significant morbidity (Whitehouse et al., 2002) and is the most common reason for unplanned readmission to hospital (Merkow et al., 2015). At worst, SSIs can even result in death (Coello et al., 2005). Ultimately, they can have major cost implications for health services (Jenks et al., 2014). Reduction of SSI is therefore of high priority to patients and to health services, and in the UK rates are routinely monitored for audit purposes in most National Health Service (NHS) Trusts. Consequently, it is essential that SSIs are accurately identified and measured for effective clinical governance and for research evaluating interventions to minimise SSI.

Commonly used tools to identify SSI include the Centres for Disease Control and Prevention (CDC) criteria and the ASEPSIS grading scale (Horan et al., 1992; Wilson et al., 1986). These tools, however, were developed primarily for use in-hospital, in circumstances when most patients had post-operative hospital stays of several days. Experienced staff are required to complete the tools, yet they do not identify SSIs consistently (Gibbons et al., 2011; Wilson et al., 2004). Problems with quantifying accurate incidence arise because many infections take time to develop and over 70% of SSIs may occur after the patient is discharged from hospital (National Institute for Health and Clinical Excellence, 2013; Petherick et al., 2006; Wilson et al., 2013). This proportion is likely to increase as surgical techniques become less invasive and hospital stays get shorter. Post-discharge assessment of SSI by healthcare professionals is possible but relies on extensive resources and is expensive, requiring additional appointments to review the wounds (Tanner et al., 2009). One solution to improving post-discharge assessment is to use patient reported outcome measures, i.e. tools completed by patients themselves. Indeed, patient-reported modifications of the CDC criteria and ASEPSIS scale have been developed and are in use (Public Health England, 2013; Wilson et al., 2013), yet these questionnaires were based on clinical and microbiological perspectives and were designed to capture similar information as in-hospital assessment. They have lacked patient input and have not been formally validated (Petherick et al., 2006). There is, therefore, a need to address these issues and design a measure for SSI that is appropriate for use after patients have been discharged from hospital. A suitably developed and validated tool for patient completion is needed, as well as a post-discharge assessment tool for healthcare professional use. However, the use of separate tools (i.e. for patient and observer completion) to assess the same outcome may introduce the risk of a different underlying construct being measured. A single measure that could be used by both patients and observers would ensure that the content validity is the same regardless of who is completing it. Such a measure may have potential applications in routine surveillance, research and clinical audit (Gibbons et al., 2011).

The aim of this study, therefore, was to develop a reliable single measure for SSI that can be used after the patient has been discharged from hospital and can be completed by the patient themselves and/or an observer.

## Methods

### Study design

This was a mixed methods study conducted in three phases using an existing framework for developing patient-centred outcome measures (Sprangers et al., 1993). Phases included: Phase 1, an analysis of existing tools and interviews with patients and professionals to establish the content of the measure; Phase 2, developing questionnaire items and designing an optimal tool; and Phase 3, cognitive interviews to pre-test the tool. Subsequent psychometric and diagnostic accuracy testing of the new measure will be described elsewhere.

Ethical approval was obtained from the NHS Health Research Authority NRES Committee London – Camden & Kings Cross (14/LO/0640). Written informed consent was obtained from all patients and professional participants.

### Phase 1: establishing the content of the measure

#### Analysis of existing tools

A detailed analysis of the most commonly used tools (definitions and grading scales) for SSI assessment, identified by a previous systematic review (Bruce et al., 2001), was undertaken. The purpose was to ascertain relevant signs, symptoms and criteria for defining SSI to include in the new measure. Tools studied were the Public Health England (PHE) clinical criteria for SSI (based on the CDC criteria) and surgical wound healing post-discharge questionnaire for patients (Public Health England, 2013), and the ASEPSIS grading scale (Wilson et al., 1986) and associated post-discharge patient questionnaire (Gibbons et al., 2011; Wilson et al., 1990). Individual criteria or questionnaire items and their response categories were extracted and recorded verbatim. Criteria and items were grouped into SSI ‘domains’ based on the sign, symptom or intervention to manage infection. Grouping was performed by four authors (BCR, JMB, AN and RM) using methods for categorising health domains described previously (Macefield et al., 2014).

#### Interviews with patients and professionals

Semi-structured interviews with patients with experience of SSI (n = 9) and professionals involved in post-surgical care (n = 10) were conducted to explore SSI signs, symptoms and interventions for managing them, and to identify new domains not covered by the existing tools. Views on the existing PHE and ASPESIS tools were collected by asking participants to complete them during interviews and asking their opinion on their suitability, relevance and ease of completion. Pre-designed interview topic guides were used to cover these objectives. Interviews were conducted by one researcher (AN), were audio-recorded and transcribed in full. Details on participant sampling are provided below.

### Phase 2: designing the measure: ‘operationalisation’ of domains and item development

Findings from Phase 1 were used to design a provisional new measure. Each of the SSI domains were operationalised into a questionnaire item according to standard processes (Sprangers et al., 1993). Items were initially worded in lay language. Where the underlying domain could be described with a medical term, this word was included at the end of the item in parentheses. Medical terms were selected from existing tools or by the study team.

The single measure was designed as a questionnaire with two grammatical variations; one using the first-person context, i.e. ‘your wound’, appropriate for patient use, and another using the third-person context, i.e. referring to ‘the wound’, to be appropriate for observer completion.

### Phase 3: pre-testing the measure

The provisional new measure was pre-tested in cognitive interviews. Patient participants (n = 28) who had undergone surgery were purposefully sampled (see below). Professional participants (n = 14) were recruited from primary care and secondary/tertiary care teams involved in surgical wound care. Patients were asked to complete the measure in relation to their own wound. Professionals were asked to use an example of a recent patient or a hypothetical case. Cognitive interviews assessed face validity, comprehension, suitability and acceptability. Interviews were conducted face-to-face with a researcher (RM or TM) at participants’ homes or places of work.

Participants were observed and asked to ‘think aloud’ while completing the questionnaire. Interviewers used probes to explore items further, such as “What does that word mean to you?” or “What do you interpret that word to mean?” (Beatty and Willis, 2007). Patients’ and professionals’ views on the inclusion of the medical terms used at the end of the items were explored. Participants were specifically asked about their understanding and perception of the medical description to ensure that the lay wording was an accurate and appropriate interpretation. Interviews were audio-recorded and summarised in descriptive memoranda.

### Participants and sampling

Eligible patients had undergone abdominal general surgery or caesarean section from two participating UK hospital Trusts. Patients were identified and approached by research nurses and surgical trainees (NB, RB and DM) while in hospital. Phase 1 sampling was restricted to patients with confirmed or suspected SSI, identified before discharge or on readmission to hospital. Phase 3 sampling included patients who had undergone surgery within the previous 30 days. Patient details were communication to other members of the study team (AN, RM or TM) to contact and arrange subsequent interviews. Healthcare professionals involved in post-surgical care from the participating hospitals were approached directly by members of the study team (AN and RM).

Participants were purposively sampled to include a range of sociodemographic characteristics and types of surgery, and a range of clinical staff and expertise. Sampling and analysis for pre-testing the measure (Phase 3) was carried out as an iterative process until no new themes emerged.

### Data analyses

*Phase 1*: A descriptive table was used to map original items from the source documents (existing clinical tools and patient questionnaires) to the identified SSI domains (Table 1). Interview data were analysed using an inductive approach. Data were coded and grouped into similar themes (thematic content analysis) (Braun and Clarke, 2006). A descriptive account of the common identified themes was generated, including signs and symptoms of wound infection, follow-up care and treatment required, commonalities across specialties and feedback on the current measures of SSI. The account concluded with a summary of points to consider when developing the new measure.

**Table 1.** Identified SSI domains and mapping of criteria and items from existing tools.

SSI domain	Criteria / Item from existing measure	Source (existing tool)
Wound healing	Have all of these wounds healed without any problem at all?	ASEPSIS PQ
	Have you had any problems with the healing of your wound?	PHE PQ
Wound heat	The area around the wound felt warmer/hotter than the surrounding skin	PHE PQ
	Heat	PHE CDS
Wound redness	Has the wound been red?	ASEPSIS PQ
	Redness or inflammation spreading from the edges of the wound	PHE PQ
	Erythema	ASEPSIS CDS
	Redness	PHE CDS
Wound discharge	Has the wound discharged clear yellow fluid? Has the wound discharged pus?	ASEPSIS PQASEPSIS PQ
	Purulent drainage	PHE CDS
	Was there any discharge or leakage of fluid from any part of the wound? If yes, was it either Clear or blood stained? Yellow/green (pus)? Other? - please specify	PHE PQ
	Serous discharge/serous exudate Purulent exudate	ASEPSIS CDSASEPSIS CDS
Layers separating - spontaneous	Has the wound broken open?	ASEPSIS PQ
	The edges of any part of the wound separated or gaped open	PHE PQ
	Separation of deep tissues	ASEPSIS CDS
	Incision spontaneously dehisces [or opened by surgeon]	PHE CDS
Wound swelling	The area around the wound became swollen	PHE PQ
	Localised swelling	PHE CDS
Wound pain	Pain or soreness in addition to the discomfort experienced following the operation	PHE PQ
	Localised pain and tenderness	PHE CDS
Fever	Fever (temperature 38ºC or more)	PHE CDS
Contact with healthcare professional	If you saw a healthcare worker because of these symptoms, please indicate who you saw from the list (GP/district nurse/midwife/doctor or nurse at the hospital/other – please specify)	PHE PQ
Dressing needed	Has a district nurse had to dress the wound?	ASEPSIS PQ
Antibiotics needed	Have you been given antibiotics for wound infection?	ASEPSIS PQ
	Have you been prescribed antibiotics for an infection in the wound? If yes, who prescribed them?________	PHE PQ
	Antibiotics prescribed	ASEPSIS CDS
	Antibiotics prescribed by GP for SSI (patient reported only)	PHE CDS
Layers separating - deliberate	Incision opened by surgeon [or spontaneously dehisces]	PHE CDS
Hospital admission	Have you been admitted to hospital elsewhere?	ASEPSIS PQ
	Have you been readmitted to hospital with an infection of the surgical wound? To the hospital at which the operation was carried out? To another hospital?	PHE PQ
Drainage needed	Drainage of pus under local anaesthesia (including vac therapy)	ASEPSIS CDS
	Purulent drainage	PHE CDS
Wound cleaning	Has the wound been opened and cleaned under general anaesthetic in hospital?	ASEPSIS PQ
	Debridement of wound (general anaesthesia)	ASEPSIS CDS
	Purulent drainage	PHE CDS
Abscess	Has a doctor opened/drained an abscess?	ASEPSIS PQ
	Abscess or other evidence of infection found during a re-operation, by radiology or histopathology examination	PHE CDS
Microbiology	Did any healthcare worker take a sample from your wound to send to the laboratory?	PHE PQ
	Aspirated fluid/swab of surgical site yields organisms and pus cells are present	PHE CDS
	Isolation of bacteria	ASEPSIS CDS
	SSI causative micro-organisms	PHE CDS
Prolonged hospital stay	Stay as inpatient prolonged over 14 days	ASEPSIS CDS
Smell	–	–

CDS, clinical data sheet; GP, general practitioner; PHE, Public Health England; PQ, patient questionnaire; SSI, surgical site infection.

*Phase 3*: Descriptive memoranda from the cognitive interviews included key findings or problems and suggestions for modifications and improvements to the provisional measure; changes to items, response categories, formatting, instruction and layout. Obvious issues or repeated suggestions were considered by the study team and edits were made in a new version of the measure. Data were analysed iteratively so that items could be modified, added or deleted to reflect emerging findings and allow further exploration of modifications in subsequent interviews.

At regular intervals throughout Phases 1–3, findings and versions of the measure were circulated to the immediate study team for comment and suggestions, and to the wider study team and collaborators before a large study meeting during Phase 2.

## Results

### Phase 1: establishing the content of the measure

#### Analysis of existing tools

A list of 42 items was generated from the PHE criteria, ASEPSIS grading scale and associated patient questionnaires. These were categorised and grouped to 18 domains; eight measuring SSI signs and symptoms and ten measuring wound management interventions (Table 1).

#### Interviews with patients and professionals

Participant demographics for the Phase 1 interviews are shown in Table 2. Patients (n = 9) had confirmed or suspected SSI after a range of gastrointestinal (GI) surgeries and professionals (n = 10) had various roles from GI, obstetric and paediatric specialties.

**Table 2.** Phase 1 participants: establishing the content of the measure.

		Professionals (n = 10)	Patients (n = 9)
*Gender*	Female	6	4
	Male	4	5
*Role*	Consultant	5	
	Midwife	1	
	Nurse	1	
	Registrar	3	
*Specialty*	Obstetrics	4	
	Paediatrics	3	
	Stoma care	1	
	Upper GI	2	
*Type of surgery*	Upper GI benign		2
	Upper GI cancer		1
	Lower GI benign		3
	Lower GI cancer		1
	Appendicectomy		2
*Wound infection*	Confirmed		2
	Suspected		1
	Absent		2
	Missing data		4

GI, gastrointestinal; GP, general practitioner.

Interview data supported findings from the analysis of the existing measures. The following signs and symptoms were described as being indicative of infection: cellulitis or redness around the wound; discharge of pus; tenderness, pain or soreness of the wound; breakdown/opening of the wound; feeling generally unwell (often associated with a temperature or fever) and occasionally tachycardia (in two interviews with staff member in obstetrics); hot wound; abscess; swelling; and raised white blood cell count. The first three signs and symptoms were mentioned more frequently, without further questioning or prompting. Wound management care and interventions included: prescription of antibiotics; wound drainage; dressing changes; cleaning of wound; taking swabs/blood/imaging investigations; observation; consulting colleagues. It was possible to map interview data to the 18 domains identified in the existing tools. In addition, however, interview data identified ‘smell’ as a domain not currently measured in existing SSI tools. This was described by both patients and professionals.

Analysis of the existing tools and interviews therefore resulted in a total of 19 domains identified domains to consider for inclusion in the new measure (Table 1).

Views on the current SSI tools and issues to consider for the new measure included simplification and use of lay language, reducing the subjectivity of items and the need to distinguish varying SSI severity in the response categories. Confusion and misunderstanding emerged with some words used in existing tools including discharge, pus, abscess and antibiotics. There was a common tendency to talk about wound healing rather than wound infection. Filter questions often gave rise to responses that were contradictory. For example, some participants responded that they had no problems with the healing of their wound (in answer to a filter question on one measure) yet responded that they had experienced some of the symptoms when asked to complete the rest of the measure.

### Phase 2: designing the measure

A provisional new measure was designed based on the identified domains and findings from Phase 1. ‘Microbiology’ and ‘Prolonged hospital stay’ were excluded domains as they were considered to be inappropriate for a measure completed by a patient or observer after discharge and because such information is more suitably obtained from hospital records.

### Phase 3: pre-testing the measure

Interviewees were patients (n = 28) and healthcare professionals (n = 14) with a range of sociodemographics and expertise (Table 3). The mean time since patients’ surgery was 46 days (range, 6–208 days). Interviews lasted on average 27 min (range, 13–52 min).

**Table 3.** Phase 3 participants: pre-testing the measure.

		Professionals (n = 14)	Patients (n = 28)
*Gender*	Female	10	11
	Male	4	17
*Age at time of interview (years)*	21–30	0	1
	31–40	7	2
	41–50	3	2
	51–60	3	6
	>60	1	17
*Role*	Midwife	3	–
	Hospital/Research nurse	3	–
	Practice/Community nurse	1	–
	Surgical trainee	4	–
	GP	3	–
*Specialty*	General practice/community	4	–
	Obstetrics	3	–
	Upper/Lower GI surgery	6	–
	Intensive care	1	–
*Length of time qualified*	<10	1	–
	10-20	7	–
	>20	6	–
*Time since surgery (weeks)*	<1	–	1
	1–2	–	2
	2–4	–	9
	>4	–	16
*Type of surgery*	Upper GI	–	9
	Lower GI	–	10
	Caesarean	–	3
	Hernia repair	–	6

Participants’ views on including medical terms alongside lay language in a single measure were divergent. For example, some professionals raised concerns about including medical terms in a questionnaire intended for patients because of the potential for generating worry or confusion, or that patients might be prompted to look up words on the Internet and see distressing images of serious cases. Generally, however, patients reported that the inclusion of medical terms in parentheses was acceptable with the majority paying attention only to the lay wording. Two patients reported that they might look up the medical term on the internet out of curiosity.

During pre-testing, interviews highlighted that the initial lay language description for some items had oversimplified the intended domain; the oversimplification only became apparent because a medical term had been included in parentheses. For example, the item intended to assess debridement of the wound (i.e. the medical removal of dead, damaged or infected tissue) was initially phrased ‘Has your wound been cleaned out? (debridement)’. However, pre-testing found that different responses were given from participants who read the lay language compared to those who read and understood the medical term. Some participants responded ‘yes’ to this item if the wound had been washed with saline solution or ‘superficially’ cleaned, whereas others responded ‘yes’ only if dead or damaged tissue had been removed from the wound. Therefore, this item was modified to ‘Has your wound been scraped or cut to remove any unwanted tissue? (debridement)’. Interviews also identified items where the medical term as well as the lay description needed revising to accurately measure the intended domain. For example, the item initially phrased ‘Have the edges of any part of the wound separated? (dehiscence)’ was revised to ‘Have the edges of any part of the wound separated on their own accord? (spontaneous dehiscence)’ to distinguish it from interventions where the wound was opened up intentionally by a doctor or nurse. These examples demonstrate that the inclusion of medical terms alongside lay language was beneficial during the development of the measure because it served as a grounding for the intended underlying construct.

**Figure 1.**
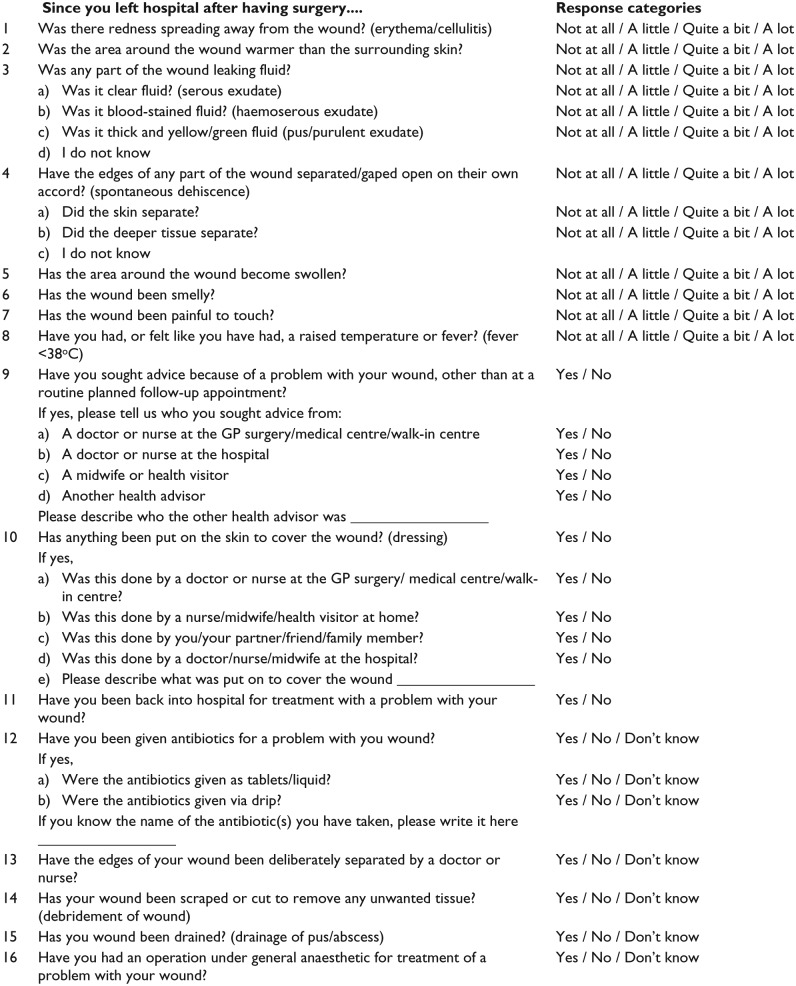
Final version of the SSI measure after pre-testing. Footnote: The version shown is for patient completion (with items written in first-person context).

The measure was modified throughout the pre-testing phase and eight versions were tested in subsequent interviews. Modifications included changes to the wording and structure of items, formatting, instructions and response categories. The final version consisted of 16 items, with nine including medical terms (Figure 1). Items relate to: (1) patient-reported signs or symptoms potentially indicative of SSI; and (2) patient-established information of wound care management and clinical interventions for treating SSI.

## Discussion

This study developed a single measure for SSI assessment that can be completed after a patient has been discharged from hospital. It has been shown to be comprehensive, easy to complete and suitable for use by patients and observers (in this example, healthcare professionals). It is advantageous for establishing information about SSI signs, symptoms and clinical interventions, irrespective of where the patient has received care during the SSI follow-up period. Describing items in lay language and with medical terminology was found to reduce the risk of misinterpretation and ensured that the intended underlying domains were measured. The measure will now undergo full psychometric testing and assessment of diagnostic accuracy to determine its suitability for use in trials or post-discharge surveillance studies.

Existing commonly used measures for SSI are based on long-standing tools developed over 20 years ago. They were primarily designed for clinical use and did not include patients’ views during development; their adaptations as patient reported questionnaires were done with little reported validity and reliability testing (Petherick et al., 2006). The new measure has been developed using thorough methodology and followed an established framework for the development of outcome measures (Sprangers et al., 1993). Mixed methods, including an examination of existing tools and interviews with patients and professionals, were used to inform the content of the measure and pre-test its acceptability. The current “dual-completion” (patient and observer) measure is a novel approach to outcome assessment, which traditionally employs multiple tools that have been developed separately and are used separately by patients and professionals. There are, however, some limitations to this study. First, interviews were conducted with patients and professionals from a small number of surgical specialties where rates of SSI are known to be higher than others. Generalisation to other surgical categories is limited and we do not know how the questionnaire will perform in a wider population. The suitability of the measure for assessing SSI in other specialities, for example, orthopaedics, is unknown. Further work is required to validate the usefulness of the measure in a larger sample and wider population. Second, observers in the context of this study referred to healthcare professionals and completion by other observers (for example, carers) was not tested. The measure also has cultural limitations, and further testing of the wording and lay language descriptions in a more diverse population may be warranted. Third, the measure has been developed for use as a postal questionnaire. An electronic adaptation that could implement filter questions and be completed and returned as an online survey might have advantages for minimising the amount of missing data. Finally, the measure has been designed for use after hospital discharge. If the measure is used during the initial hospital stay some of the items are redundant (e.g. ‘Have you been back into hospital for treatment of a problem with your wound?’).

The measure is currently being tested for validity and reliability in a larger sample of patients (approximately 400) undergoing abdominal surgery. How the responses from a completed questionnaire may be used to diagnose the presence/absence or type of SSI will be investigated in the analysis of the validation study and findings will be reported separately. Following validation and diagnostic accuracy testing, the suitability of the measure for collecting SSI outcome data for research and routine surveillance can be evaluated. Future research using the new measure is intended in randomised controlled trials of interventions to reduce SSI.
